# GLUT4 dispersal at the plasma membrane of adipocytes: a super-resolved journey

**DOI:** 10.1042/BSR20230946

**Published:** 2023-10-20

**Authors:** Angéline Geiser, Shannan Foylan, Peter W. Tinning, Nia J. Bryant, Gwyn W. Gould

**Affiliations:** 1 Strathclyde Institute of Pharmacy and Biomedical Sciences, University of Strathclyde, Glasgow, U.K.; 2 Department of Biology, University of York, Heslington, York, U.K.

**Keywords:** adipocytes, glucose transport, insulin, microscopy

## Abstract

In adipose tissue, insulin stimulates glucose uptake by mediating the translocation of GLUT4 from intracellular vesicles to the plasma membrane. In 2010, insulin was revealed to also have a fundamental impact on the spatial distribution of GLUT4 within the plasma membrane, with the existence of two GLUT4 populations at the plasma membrane being defined: (1) as stationary clusters and (2) as diffusible monomers. In this model, in the absence of insulin, plasma membrane-fused GLUT4 are found to behave as clusters. These clusters are thought to arise from exocytic events that retain GLUT4 at their fusion sites; this has been proposed to function as an intermediate hub between GLUT4 exocytosis and re-internalisation. By contrast, insulin stimulation induces the dispersal of GLUT4 clusters into monomers and favours a distinct type of GLUT4-vesicle fusion event, known as fusion-with-release exocytosis. Here, we review how super-resolution microscopy approaches have allowed investigation of the characteristics of plasma membrane-fused GLUT4 and further discuss regulatory step(s) involved in the GLUT4 dispersal machinery, introducing the scaffold protein EFR3 which facilitates localisation of phosphatidylinositol 4-kinase type IIIα (PI4KIIIα) to the cell surface. We consider how dispersal may be linked to the control of transporter activity, consider whether macro-organisation may be a widely used phenomenon to control proteins within the plasma membrane, and speculate on the origin of different forms of GLUT4-vesicle exocytosis.

## Introduction

### GLUT4 trafficking

Glucose plays a pivotal metabolic role in most mammalian cells. Its diffusion from the bloodstream into cells relies on a family of 14 glucose transporters, known as GLUTs, that are expressed in a tissue-specific manner [1]. Following isolation of GLUT1 cDNA in 1985 [2], a further 13 members of the GLUT family were identified: GLUT2 to 12, HMIT, and GLUT14 [1,3–6].

**Figure 1:**
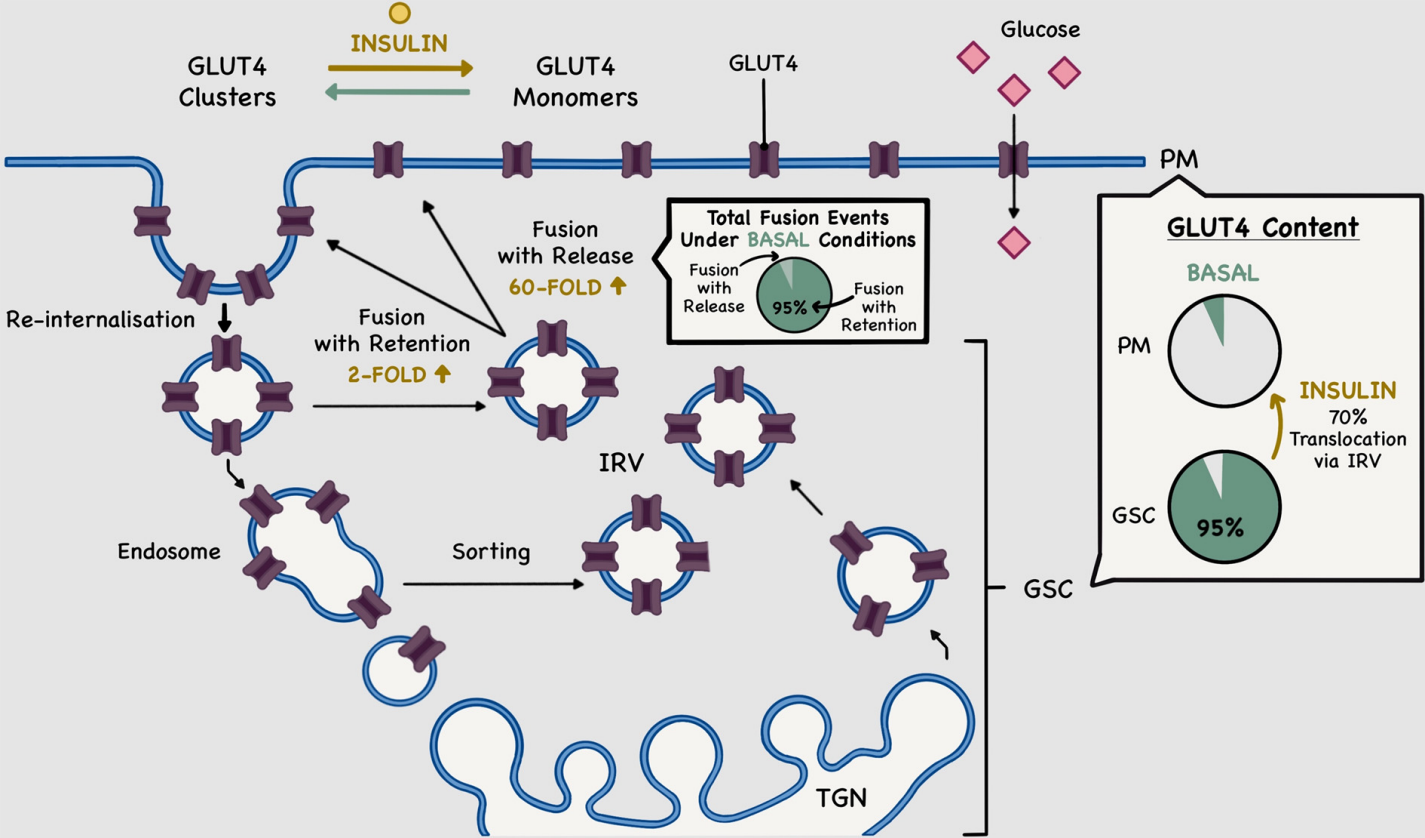
Model of GLUT4 trafficking to, and dispersal within, the plasma membrane in adipocytes In adipocytes, insulin regulates GLUT4 translocation to, and dispersal within, the plasma membrane (PM). Under basal conditions, 95% of cellular GLUT4 transporters (shown as purple structures) accumulate in GSC, including the *trans*-Golgi network (TGN), endosomes, and insulin responsive vesicles (IRV). Upon insulin stimulation, up to 70% of the sequestered GLUT4, primarily in IRV, translocate to and fuse with the PM. In terms of exocytosis, in the basal state, 95% of all fusion events happen in a fusion-with-retention manner, upon which PM-fused GLUT4 molecules are retained at their site of fusion forming clusters. In the presence of insulin, the number of fusion-with-retention events increases by 2-fold. Fusion-with-release events are increased by 60-fold. GLUT4 transporters therefore primary undergo exocytosis through the fusion of IRV and release into the PM as monomers, leading to increased and more efficient glucose (pink) uptake into the cell. Upon insulin removal, GLUT4 molecules are re-internalised and recycled back into the GSC. For details, refer to text and [69].

In 1939, Einar Lundsgaard first observed an insulin-mediated increase in glucose uptake into cat skeletal muscle [7,8]. This phenomenon was subsequently shown, first in adipocytes [9–11] and later in muscle cells [12,13], to rely on a specific trafficking system that involves the insulin-dependent translocation of glucose transporters - subsequently identified as GLUT4 - from intracellular compartments, now defined as GLU4 storage compartments (GSC) [14], to the plasma membrane (PM) (Figure 1). In both adipose and muscle tissues, GLUT4 is the main glucose transporter to undergo insulin-stimulated translocation [15–19].

In adipocytes, it is now established that in the absence of insulin 95% of cellular GLUT4 transporters accumulate in GSC, which includes the *trans*-Golgi network (TGN), endosomes, and ‘insulin responsive vesicles’ (IRV) [20–22]. Upon insulin stimulation, up to 70% of the sequestered GLUT4 undergoes exocytosis to the PM [20,23,24]. IRV are transported along cytoskeletal elements, facilitating their final tethering, docking, and fusion to the PM. As a consequence, GLUT4 levels at the PM increase with a concomitant increase in glucose entry into the cells [21,22,25,26]. Upon insulin removal, most of the PM-localised GLUT4 are re-internalised into IRV [25] (Figure 1). Failure of GLUT4 to undergo insulin-stimulated translocation to the PM represents a major manifestation of insulin resistance in type 2 diabetes [25]. This has in turn driven extensive efforts to understand the itinerary of GLUT4 trafficking within cells, and to understand how its delivery to and from the PM is regulated. The complexity of GLUT4 trafficking is exemplified by the number of proteins identified as regulators of different aspects of intracellular GLUT4 storage, the levels of many changing in type 2 diabetes, and by the difficulties in unravelling the pathways involved in its endocytotic trafficking [27]. These areas have been the subject of many excellent reviews [21,22,26,28–32].

### GLUT4 trafficking revealed by GFP

The discovery and subsequent use of green fluorescent proteins (GFP) as localisation markers for sub-cellular structures [33,34] fundamentally altered the scope of questions which could be addressed by cell biologists. In 1996, GLUT4-GFP was expressed in 3T3-L1 adipocytes, allowing for real-time observation of GLUT4 insulin-mediated translocation to the PM for the first time [35]. Subsequently, a hemagglutinin (HA)-tag was added to the first exofacial loop of the GLUT4-GFP construct and expressed in primary rat adipocytes. Using confocal microscopy, this allowed distinction of intracellular and PM-fused GLUT4 populations [36]. These studies laid the foundations for many future projects using fluorescence microscopy to investigate GLUT4 dynamics, and the HA-GLUT4-GFP construct became widely used in studies of GLUT4 traffic.

The main site of GLUT4 function is at the PM. While the steps of GLUT4 trafficking to the PM have been dissected and insulin established as the main regulator, less is known about the dynamics of GLUT4 within the PM. 30% of human genes encode membrane proteins [37] and given that one of the most fundamental functions of the PM is the transport of materials/signals into and out of the cell, complex cellular mechanisms likely regulate the activity and location of membrane proteins such as GLUT4. To study the behaviour and regulatory mechanisms of individual or group of molecules within the PM, structural and dynamic information is required on length scales, below the resolving power of traditional light (optical) microscopy.

**Figure 2:**
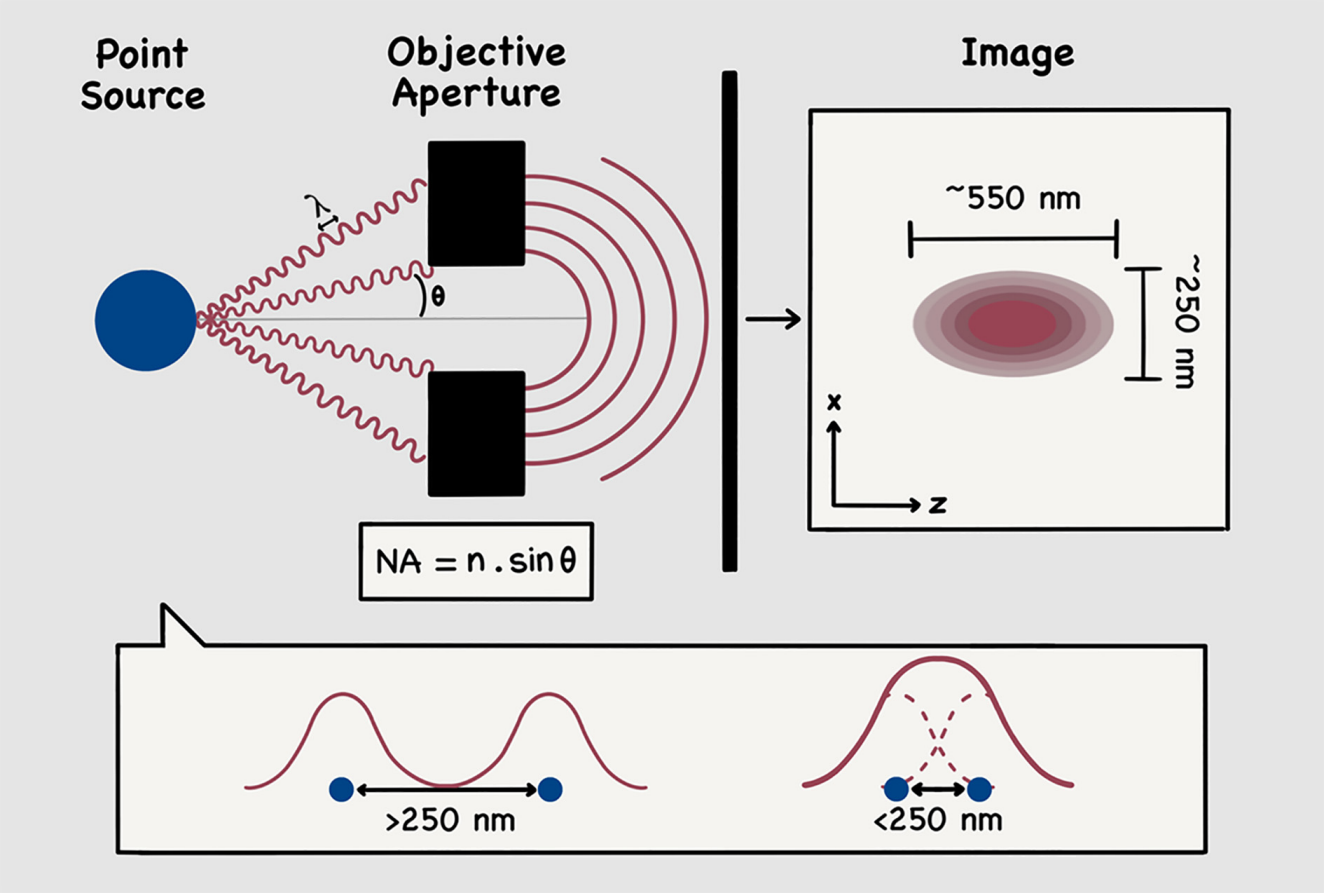
Diffraction-limited resolution of light (optical) microscopy As it passes through the objective aperture, the emitted light emanating from a single point source (blue) is diffracted into a series of concentric rings, ultimately producing an image that is broadened and lacks the sharpness of the original illuminated details. Using a conventional light microscope with a high numerical aperture (NA), the spatial resolution of the image point source is limited to approximately 250 nm laterally (*x*) and 550 nm axially (*z*), depending on the used wavelength (λ). While multiple point sources separated by a distance larger than this resolution limit appear as two separate entities in the image, if positioned at a closer distance, a single entity will be observed. NA = *n*.sinθ, where *n* is refraction rate between the point source and the objective (Air: *n*=1, Immersion oil: *n*=1.52) and θ is the half-angle of the maximum cone of light that can enter the objective lens aperture.

The ability of optical microscopes to distinguish individual structures from one another is indeed physically limited, as first described in 1873 by Ernst Karl Abbe [38]. Due to its wave nature, the light collected through an objective lens aperture which emanates from a single point source, such as a fluorophore, ultimately produces an image that is broadened into a series of concentric rings and lacks the sharpness of the original details within the illuminated specimen (Figure 2). Following on from Abbe's work, it was established that light microscopes cannot distinguish between two objects closer than λ/2NA, where λ is the wavelength of light used to image the specimen and NA is the numerical aperture of the imaging lens. More specifically, NA is defined as *n*.sinθ, where *n* is the refractive index of the medium in which the light propagates between the point source and the objective (Air: *n*=1, Immersion oil: *n*=1.52) and θ is the half-angle of the maximum cone of light that can enter the objective lens aperture (Figure 2). Accordingly, the only way to increase the resolving power of an imaging system is through some combination of reducing λ or increasing NA, both of which have physical limitations.

**Figure 3:**
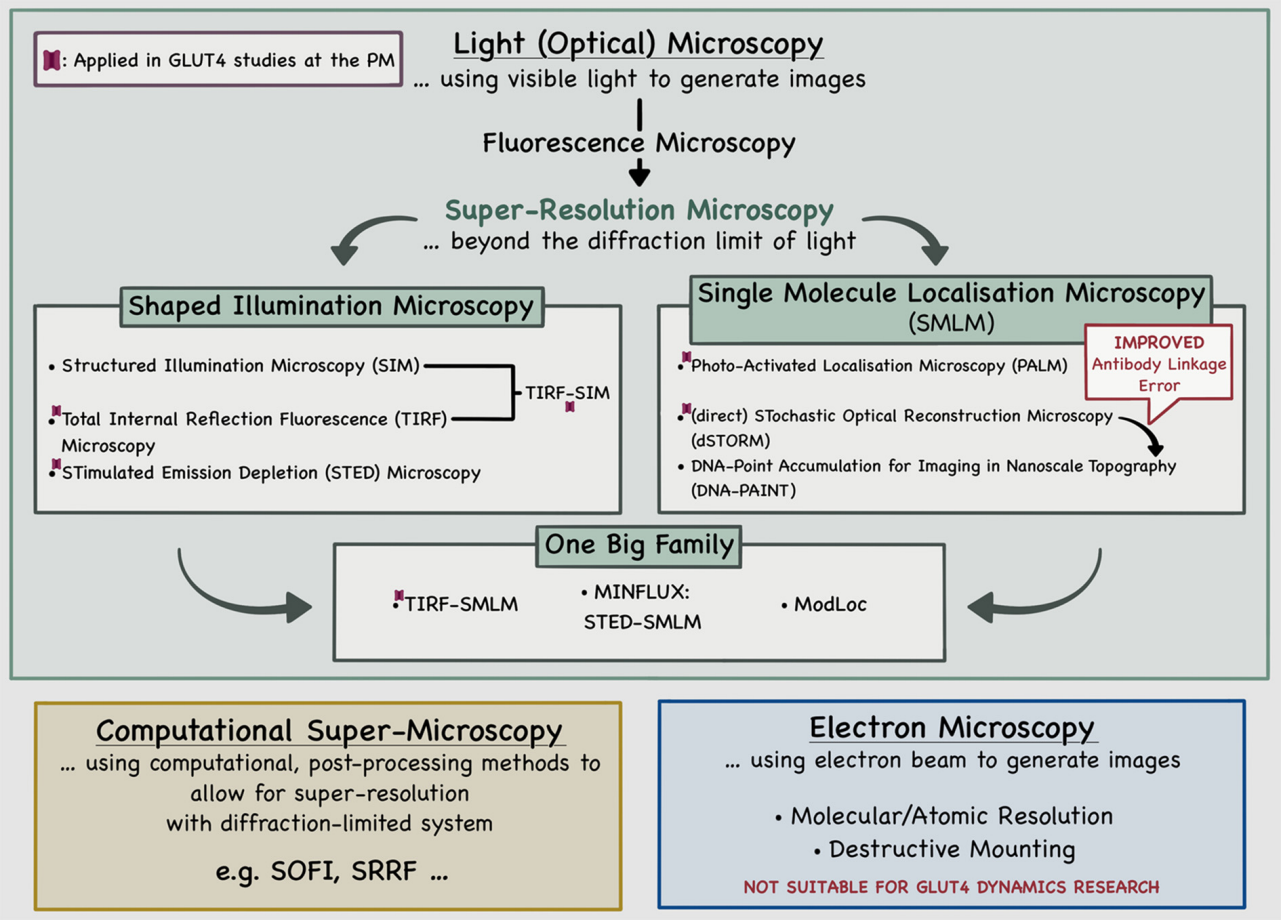
Super-resolution microscopy approaches and GLUT4 studies at the plasma membrane This figure illustrates an overview of super-resolution light (optical) microscopy in the GLUT4 field and highlights (purple structures; GLUT4) which techniques have been applied to elucidate the mechanism of GLUT4 behaviour *within* the PM: TIRF [69]; TIRF-SIM [67,68] STED [67]; PALM & TIRF-SMLM [71]; dSTORM [72–74]. TIRF, SIM, STED, and TIRF-SIM, have also all been deployed to study aspects of GLUT4 intracellular trafficking and delivery to the cell surface; see for example [60–68]. Note that in dSTORM, requirement of fluorescently labelled antibodies decreases the quantitative localisation accuracy of targeted molecules due to their size, known as the antibody ‘linkage error’. This error is improved (minimised) in DNA-PAINT. Computational super-microscopy is included to highlight that these relatively new approaches offer potential to further enhance our resolution and understanding of GLUT4 dynamics using diffraction-limited systems. The reader is referred to several excellent reviews to informatively select such methodologies for their purposes [39–44]. For details, refer to text.

Microscopy techniques which do not rely on illumination with light, such as electron microscopy, do not suffer from the limit outlined above. However, while electron microscopes can offer molecular and even atomic resolution [24], the need for specimen fixation and destructive mounting means that biologists still favour fluorescence microscopy, despite the limit on resolution, which allows live imaging of specimens in atmospheric conditions and non-invasively. In the past 30 years, there has been a concerted effort to surpass the diffraction limit of light, resulting in a new family of fluorescence imaging, known as super-resolution microscopy. After an overview of these approaches, we consider in this review their impact on our understanding of GLUT4 (Figure 3).

## Super-resolution microscopy – a new era of microscopy

Super-resolution microscopy, or nanoscopy, bypasses the diffraction limit imposed by the physical properties of light by utilising phenomena in optical physics and molecular chemistry to resolve structures previously only observable with electron or atomic force microscopy, but without the rigorous and often destructive sample preparation required by these methods. The range of light (optical) techniques which fall under the umbrella of super-resolution microscopy is extensive, and ever-growing. However, there are several useful review articles which enable researchers to informatively select the most appropriate super-resolution method for their purposes [39–44]. Broadly, the field of super-resolution microscopy can be split into two categories: techniques which rely on shaped illumination, and localisation-based methods.

### Shaped illumination techniques

The first category of super-resolution microscopy approaches includes techniques which rely on altering the excitation beam path and therefore how it is projected onto the studied specimen. The oldest approach within this family is termed total internal reflection fluorescence (TIRF) microscopy. This technique improves the axial resolution of diffraction limited systems by employing the evanescent byproduct of a laser beam undergoing total internal reflection, to excite exclusively fluorophores within a few hundred nanometres of the specimen-substrate interface [45]. As will be briefly discussed below, this approach is widely used to study GLUT4-vesicles dynamics (Figure 3).

A further technique is structured illumination microscopy (SIM), which includes 2D-SIM, a method where typically nine spatially altered illumination grating patterns are sequentially applied into the excitation beam path. Subsequent computational processing of images acquired using this approach allows for a 2-fold improvement in lateral resolution, beyond the diffraction limit of the imaging objective lens [46]. To extend this concept to volumetric imaging, SIM can also be combined with light sheet microscopy (LSM). While LSM is a diffraction-limited technique, it has provided great biological insight over the years [47]. By decoupling excitation and illumination paths, this multi-objective, high-resolution shaped illumination method allows fine optical sectioning of thick biological samples without the need for physical sectioning. Super-resolution LSM-SIM, also known as Lattice LSM, therefore uses the diffraction grating of SIM, alongside a lightsheet illumination source to achieve the spatial resolution of SIM through depth [47].

Finally, a much improved lateral spatial resolution, on the order of 30-50 nm, can be achieved using stimulated emission depletion (STED) microscopy [48,49]. This method relies on having two laser sources, a conventional Gaussian excitation beam and a longer wavelength masked ‘donut-shaped’ depletion beam which overlap, and force emitting fluorophores within the profile of the depletion beam into a dark ground state. Therefore, only fluorophores within the unmasked region of the excitation beam are detected, giving rise to sub-diffraction limit resolution. Consequently, this approach is considered as somewhat less gentle.

### Single molecule localisation microscopy (SMLM) techniques

The second family of super-resolution techniques, namely single molecule localisation microscopy (SMLM) methods, was developed on the basis that the precise localisation of a single fluorophore within a studied image can be established by mapping its intensity to a Gaussian function, the peak of that function being directly related to the localisation where the most photons are produced. However, as previously explained, due to the diffraction limit of light, when viewed under a microscope, the size of the image, as well as the intensity distribution, of the fluorophore will appear larger than its true size, limiting the spatial resolution of a conventional light microscope with a high NA to around 250 nm laterally and 550 nm in the axial direction, depending on the used λ [40]. This signifies that, if positioned at a lower distance from each other than the wavelength of the light used to image them (<250 nm), closely labelled proteins will appear as a single fluorescent entity when viewed through a microscope, leaving their precise localisation undetectable (Figure 2). SMLM methods therefore approach visualising structures below the diffraction limit by iteratively switching on and off fluorescent molecules, such that only a sparse population of single molecules are detected at any one time.

The first methods for achieving SMLM were shown with photo-activated localisation microscopy (PALM), where the specimen was imaged and subsequently bleached through depth [50], and stochastic orientation reconstruction microscopy (STORM), which used photo-switchable fluorophores to image single molecules over several thousand image frames [51]. Subsequently, the use of conventional cyanine dyes with specialised buffer solutions became increasingly popular [52], allowing for direct STORM (dSTORM), the direct nature coming from the fact that fluorescent antibody pairs are not required [53,54]. More recently, fluorescent transiently-binding single DNA strands have re-emerged as a versatile tool, allowing for improved single molecule localisation with the development of point accumulation in nanoscale topography (DNA-PAINT) [55].

### One big family

Truly, both super-resolution families do not have to remain as separate entities. An efficient means of improving the localisation precision of SMLM has been demonstrated by the concurrent use of shaped illumination methods, with TIRF microscopy lending itself well as the most popular illumination method for SMLM. Its thin, tunable depth optical range significantly reducing background fluorescence and thus increasing localisation precision. A further technique, termed ModLoc, was developed to employ structured modulation patterns, allowing for a uniform 6.8 nm axial localisation precision through several microns of a specimen [56]. The principle of STED and SMLM have also been combined to achieve isotropic resolution on the order of 1-3 nm in a technique called MINFLUX [57].

A variety of methods have also been developed as purely computational and post-processing in nature, such as super-resolution optical fluctuation imaging (SOFI) [58] and super-resolution radial fluctuations (SRRF) [59], allowing for super-resolution to be achieved using diffraction-limited systems.

Collectively, these tools open the range of structures and biological processes that can be studied. Below we consider the impact of some of these on GLUT4 biology (Figure 3).

## GLUT4 dynamics near and within the plasma membrane

The emergence of new fluorescence microscopy approaches permits investigations beyond the diffraction limit of light and have allowed further study of GLUT4 behaviour near (approaching and adjacent to the PM) and within the PM (i.e., post fusion) (Figure 3). Some key studies performed to date are reviewed here. We use the term ‘GLUT4-vesicles’ to reflect a level of ambiguity/controversy regarding which sub-population of the GSC is being studied [22].

### Insulin-stimulated GLUT4-vesicle fusion

In 2004, using EGFP-fused GLUT4, Li et al. were the first to apply TIRF microscopy to investigate the 3D mobility of GLUT4-vesicles approaching the PM in live 3T3-L1 adipocytes. Tracking GLUT4-vesicles using TIRF and single-particle analysis revealed the restricted movement of GLUT4-vesicles within a mean radius of 160 nm from the PM, suggesting the presence of an intracellular tethering matrix [60]. GLUT4-vesicles were found to exhibit a smooth continuum of 3D diffusional coefficients, indicating that despite there being several potential routes for GLUT4-vesicles to reach the PM, they appear to be organised in a continuous range of mobility and do not reflect distinct ‘classes’ of GLUT4-vesicles (see below) [60]. In the absence of insulin, similar behaviour of GLUT4-vesicles rapidly moving along the PM, periodically stopping and loosely tethering to the membrane, were also observed by applying time-lapse TIRF microscopy in primary rat adipocytes [61].

The use of a dual-coloured probe to follow GLUT4-vesicle trafficking in TIRF mode also represented a further advance, allowing quantification of fusion events as well as vesicular location. Insulin was observed to induce a 40-fold increase in the fusion of GLUT4-vesicles with the PM in 3T3-L1 adipocytes compared with basal conditions. However, it was observed that a fraction (∼15%) of GLUT4-vesicle fusion to the PM exhibited a fusion-with-retention or ‘kiss-and-run’ type event, where PM-fused GLUT4 molecules are retained at their sites of fusion before being re-internalised from the PM [62]. Lizunov et al. similarly showed that insulin regulates immobilisation, tethering, and fusion of GLUT4-vesicles with the PM in primary rat adipocytes [63].

TIRF microscopy, as well as SIM, STED, and TIRF-SIM, have all been deployed to study aspects of GLUT4 intracellular trafficking and delivery to the cell surfaces; see for example [60–68].

### Insulin-regulated GLUT4 dispersal within the plasma membrane

While the studies outlined above confirmed that insulin affects intracellular GLUT4 trafficking and fusion to the PM, in 2010, it was suggested that insulin also has an impact on the spatial distribution of GLUT4 within the PM [69]. Using multi-colour TIRF microscopy (Figure 3), the existence of GLUT4 molecules present as stationary ‘clusters’ at the cell surface was revealed. It was further suggested that a portion of GLUT4 in the PM existed as monomers. Stenkula et al. posited that these monomers derive from the exocytosis of GLUT4-vesicles involved in fusion-with-release type events, where GLUT4 transporters are shown to rapidly spread out into the PM, away from their sites of fusion. Experimentally, the authors showed that in the basal state, fusion-with-retention events represent 95% of all fusion events (Figure 1). At this point, the amounts of clustered and monomeric GLUT4 at the PM were found to be broadly similar. Insulin was observed to increase the total fusion rate of GLUT4-vesicles with the PM from 0.03 events/μm^2^/min to 0.15 events/μm^2^/min after only 2 min of stimulation. This increase was shown to be the result of the differential stimulation of both previously described modes of exocytosis [62], with a 60-fold increase on the number of fusion-with-release events against a 2-fold increase in fusion-with-retention. These boosts in fusion events could account for observed increases in both total PM-fused GLUT4 as well as clustered and dispersed GLUT4, respectively (Figure 1). Hence, suggesting that in the basal state, fusion-with-retention is the primary GLUT4 provider of the PM in the form of clusters. Insulin stimulation then triggers fusion-with-release events where a significant amount of GLUT4 is delivered to, and dispersed into, the PM. Overall, these observations demonstrated that GLUT4 proteins are distributed non-homogeneously within the PM. Nevertheless, it was established that upon insulin-stimulation the total amount of translocated GLUT4 corresponded broadly to a cellular increase in glucose uptake, suggesting that both GLUT4 populations at the PM are functional glucose transporters [69].

Considering the above data, Stenkula et al. proposed a new kinetic model to explain GLUT4 behaviour among GSC and the PM, which they based on the inclusion of specific parameters. The first being that, at the PM, insulin only affects the dispersal of GLUT4 (i.e., not its activity), and the second that the amount of GLUT4 in all GSC, apart from IRV, remains relatively constant upon insulin stimulation. This extends previous models [70] by adding GLUT4 clusters as a new quasi-compartment. Under basal conditions, PM-fused GLUT4 are mostly found to localise in this compartment, which function as intermediate hubs between GLUT4 exocytosis and re-internalisation. Upon insulin stimulation, this model considers a rapid increase in GLUT4 monomers at the PM primarily due to an increase in GLUT4-vesicle fusion, more specifically fusion-with-release events (Figure 1). Based on this new kinetic model, Stenkula et al. provided evidence for a hitherto unidentified step to GLUT4 trafficking system: insulin increases GLUT4 dispersal within the PM, from clusters to monomers, and glucose uptake becomes more efficient [69].

The GLUT4 dispersal model is readily amenable to probe using super-resolution microscopy, and different techniques have been developed to further investigate its dynamics within the PM in the presence of insulin. By transfecting adipocytes with HA-GLUT4-EOS probe and using a TIRF-PALM microscopy system (Figure 3), Lizunov et al. confirmed that insulin contributes to the transfer of GLUT4 from a clustered to a monomeric state (Figure 1). Insulin increases GLUT4 monomer dissociation from clusters, as well as decreasing the rate of GLUT4 endocytosis [71]. The spatial arrangement of HA-GLUT4-GFP within the PM of 3T3-L1 adipocytes has been studied at a single molecule level using dSTORM [72,73] (Figure 3). The studies reinforced the idea that insulin stimulates GLUT4 dispersal *within* the PM, after fusion of GLUT4-vesicles, highlighting a novel facet of GLUT4 biology with potentially important consequences. Further, our group assessed whether GLUT4 dispersal operates in cell types other than adipocytes. We ectopically expressed HA-GLUT4-GFP in either induced pluripotent stem cell-derived cardiomyocytes or HeLa cells, and in both cell-types observed insulin-stimulated GLUT4 dispersal [74]. Of note, insulin-stimulated dispersal of the transferrin receptor (TfR), was not observed in HeLa cells, supporting the hypothesis that this mechanism is unique to GLUT4; however, we will return to this point below.

### Impaired GLUT4 dispersal in insulin resistance

In 2017, Gao et al. used dSTORM to image HA-GLUT4-GFP at the PM of basal and maximally insulin-stimulated 3T3-L1 adipocytes [72]. Consistent with data from TIRF microscopy, in the presence of insulin, the authors observed a shift in the distribution of GLUT4 at the PM to a more dispersed configuration. Interestingly, experimental insulin resistance was also observed to increase GLUT4 clustering, suggesting a link between the clustering ability of GLUT4 and insulin sensitivity [72]. Similar results were observed by our group [74]. This link was further revealed when considered how GLUT4 dispersal is correlated with cell size [74,75]. Adipocytes of different sizes have been shown to exhibit distinct metabolic properties: larger adipocytes exhibit reduced insulin-stimulated glucose transport compared to smaller cells and are associated with adverse metabolic outcomes [76]. Strikingly, we observed that GLUT4 dispersal is reduced in larger cells, supporting the hypothesis that larger adipocytes are refractory to insulin challenge and further suggesting that GLUT4 dispersal is an integral facet of the cellular response to insulin. Such studies emphasise a need to understand the mechanism(s) which underpin this dispersal.

## GLUT4 dispersal machinery

It is now well established that many molecular mechanisms that regulate membrane traffic are conserved through evolution, from single cell organisms such as yeast to metazoans, and many seminal findings in the GLUT4 field have initially been made using genetically tractable systems such as *Saccharomyces cerevisiae*, *Drosophila melanogaster* and *Caenorhabditis elegans*. When heterologously expressed in *S. cerevisiae*, human GLUT4 is sequestered intracellularly, as observed in adipose and muscle cells [77]. Such observations indicate that the pathway followed by GLUT4 in insulin-sensitive cells is conserved through evolution from yeast to mammals and has allowed the use of yeast as a model system to study GLUT4 trafficking and thus potentially identify key genes involved in aspects of GLUT4 biology.

Wieczorke et al. used a strain of *S. cerevisiae* engineered to lack hexose transporters (Δhxt) and therefore unable to grow on media supplemented with hexoses as carbon source. Interestingly, the expression of mammalian (rat and human) GLUT4 in this mutant did not rescue growth on glucose [78]. Previous studies showed that, when heterogeneously expressed in *S. cerevisiae* with partially deleted endogenous glucose transporters, rat GLUT4 are retained intracellularly and do not contribute to glucose uptake [79]. However, Wieczorke et al. observed a clear redistribution of ectopically expressed human GLUT4 toward the PM in their Δhxt yeast strain [78], supporting the idea that regulatory machinery of GLUT4 within the PM may play an important role in glucose cellular uptake. A genetic screen to generate mutants of the Δhxt strain that enable the expressed GLUT4 to support glucose uptake identified the recessive mutant *fgy1-1* allele [78,80]. This allele encodes a mutant version of *EFR3*. Efr3 is a membrane protein, essential for the assembly of Stt4-containing patches at the PM. Stt4 is the yeast ortholog of the human phosphatidylinositol 4-kinase type IIIα (PI4K-IIIα), which catalyses the synthesis of phosphatidylinositol 4-phosphate (PI4P), an essential signalling lipid at the PM [81,82].

### EFR3 and PI4K-IIIα regulate GLUT4 dispersal in adipocytes

Our group tested the hypothesis that EFR3 and PI4K-IIIα play key roles in the insulin-stimulated regulation of GLUT4 at the PM, in adipocytes [73]. With a 62% sequence identity, EFR3 was shown to be evolutionarily conserved from mammals to yeast and in higher eukaryotes to have two paralogs: EFR3A and EFR3B. Both proteins contain a N-terminal cysteine rich region that encodes palmitoylation sites, allowing anchoring at the PM. Adjacent, armadillo-repeats permit protein-protein interactions [83]. In 3T3-L1 adipocytes, EFR3A was shown to have a higher expression, with mRNA levels >500-fold higher than EFR3B [73].

SiRNA knockdown of EFR3A or PI4K-IIIα significantly reduced insulin-stimulated glucose transport in 3T3-L1 adipocytes [73], and in as yet unpublished data, we have further shown that the PI4K-IIIα selective inhibitor C7 (Ximbio, P/N 153579) [84,85] also significantly reduced insulin-stimulated glucose transport (Angéline Geiser, unpublished work). Collectively, these data support the hypothesis that that EFR3A and PI4K-IIIα play a role in insulin-stimulated glucose transport in adipocytes. Given the location of EFR3A and PI4K-IIIα at the PM, we asked whether depletion of EFR3 might affect GLUT4 dispersal. We used 3T3-L1 adipocytes expressing HA-GLUT4-GFP and adopted a dSTORM approach (Figure 3) to assess the effects of EFR3A knockdown on GLUT4 dispersal in the presence and absence of insulin. siRNA-mediated knockdown of EFR3A significantly inhibited the ability of insulin to promote GLUT4 dispersal, suggesting that depletion of EFR3A is required for the transition of GLUT4 from clustered to dispersed monomers in the PM [73].

Our data are consistent with the hypothesis that EFR3A and PI4K-IIIα are critical components of a newly identified mechanism that controls insulin-stimulated GLUT4 dispersal at the surface of adipocytes. Future challenges will be to understand how this relates to GLUT4 dynamics in the PM, how the insulin signalling machinery interacts with EFR3A and PI4K-IIIα, and the role of specific phosphoinositide species in controlling dispersal.

## Unanswered questions – limitations and speculations

With the advent of both ultra-fast imaging (image capture rated of up to 10 kHz have recently been described; [86]) and super-resolution microscopy (Figure 3), the macro-organisation of membrane proteins within the PM, specifically has received much attention, enhancing understanding of the regulation oligomerisation [87–89], the assembly of mobile and non-mobile structures [88–91], the role of lipid rafts [92], and the concept of transient confinement zones [92,93]. Hence, there is an increasing understanding that the regulation of proteins within the PM can have significant implications on their function, as has been suggested by studies on GLUT4 dispersal described above. Increased understanding inevitably leads to more focused questions: how widespread is this phenomenon of dispersal, does dispersal act to regulate the activity of glucose transporters, and how are the different modes of GLUT4 exocytosis rationalised within existing models? Some studies have begun to shed light on these questions.

### Dispersal and clustering of GLUTs – size of clusters and effects on activity

Using dSTORM, Yan et al. studied the behaviour of GLUT1 at the cell surface HeLa cells [94]. Results showed that GLUT1 form clusters, suggested to be regulated by lipid rafts, the actin cytoskeleton, as well as by glycosylation of GLUT1. Clusters were observed to possess an average diameter of 250 nm on the apical membrane (i.e. medium-exposed membrane) and 137 nm on the basal surface of cells (i.e. facing the coverslip) [94]. Estimations of the number of GLUT1 in these structures revealed that the majority contained between two and four molecules, and that approximately 35% of these clusters were associated with lipid rafts. While these data provide clear evidence in favour of GLUT1 adopting a clustered state, it should be noted that these estimates are subject to the linkage error of labelling antibodies (see below) (Figure 3). Hence, the precise stoichiometry of these clusters remains uncertain. It is important to clearly state that our studies using dSTORM are subject to similar limitations, thus our reluctance to quantify numbers of GLUT4 molecules per clusters [73,74].

Nevertheless, Yan et al. considered how GLUT1 clustering may be related to its activity. Both methyl-cyclodextrin (a cholesterol-depletion agent) and sodium azide (a metabolic poison) are known to mediate a small increase in glucose transport in HeLa cells. dSTORM analysis suggested that upon treatment with these agents, the number of GLUT1 monomers increased, while the apparent diameter of clusters decreased. These results further suggest that the activation of GLUT1 might correlate with changes in the molecular organisation of its clusters [94].

While correlative, these observations provide a further potential link between GLUT dispersal and increased activity. Using classical kinetic approaches, GLUT1 oligomerisation has been implicated in the control of transport activity, but how this oligomerisation relates to clusters reported by dSTORM remains uncertain [95–97]. A driver for further consideration of this point has been provided by recent data indicating that some mutations of GLUT1-deficiency syndrome, involving reduced expression or loss of function of GLUT1 in the brain, may impact GLUT1 oligomerisation [98]. This is an exciting area of research worthy of further investigations.

Some comparison with data on GLUT4 on this point is informative. Using our dSTORM datasets for control and EFR3 knockdown adipocytes [73], we used a recently described ImageJ plug-in [99] to quantify the amount of GLUT4 molecules present at the surface of those same cells. While the knockdown of EFR3A significantly decreased GLUT4 dispersal at the PM, total levels of GLUT4 in both control and EFR3A-knockdown cells appeared to be similar, suggesting that EFR3A is not engaged in the translocation of GLUT4 to the PM [73]. Under the same conditions however, insulin-stimulated glucose transport is impaired upon EFR3A knockdown. Hence, similarly to GLUT1, these (correlative) findings further suggest that regulation of dispersal may represent a physiological control mechanism for this group of transport proteins. Further studies are needed to evaluate this hypothesis and are presently on-going in our laboratory.

### Dispersal and clustering – a behaviour of PM-resident proteins?

The question of whether other PM-localised proteins exhibit clustering/dispersal abilities is also worthy of further consideration. This was to some extent addressed by comparing the behaviour of GLUT4 and the TfR in insulin-stimulated HeLa cells [74]. While effects of insulin on clustering of GLUT4 were small (but still clearly evident), data revealed that the TfR did not appear to exhibit insulin-dependent dispersal. However, it would be wise to replicate these studies in physiologically insulin sensitive cells.

Related to this point, a fascinating recent study has provided evidence for insulin receptor incorporation into ‘dynamic clusters’ at the PM of hepatocytes and adipocytes [100], revealing a dynamic redistribution of insulin receptors into so-called ’condensates’. Biomolecular condensates are proposed to be compartments within cells into which proteins can be concentrated without being delimited by a membrane. First observed in T-cells upon activation of the T-cell receptor [101], these condensates been widely reported to act as a foci for organising signalling platforms. Studies using covalently linked probes to monitor the properties of the membrane environment around integral membrane proteins have revealed that insulin drives a significant change in the fluidity of the PM in the region of the receptor, while exerting minimal effects on the average PM fluidity [102]. These data suggest that changes in the lipid microenvironment may underscore changes in clustering behaviour. How these relate to GLUT4, and other protein clusters remains to be revealed.

### Dispersal and clustering – solving the antibody linkage issue

As previously described, super-resolution microscopy has allowed to gain substantial understanding of GLUT4 within the PM of adipocytes (Figure 3), with the identification of new GLUT4 dispersal regulatory actors. However, in this review, we also highlight multiple unanswered questions about the clustering nature of GLUT4, and how these uncertainties relate to the limitations of certain super-resolution microscopy techniques. For instance, while dSTORM generates consistent imaging datasets, the requirement of fluorescently labelled antibodies decreases the quantitative localisation accuracy of targeted molecules due to their size. As a result, each recorded localisation corresponds to much larger structures than the initial proteins of interest, artificially scaling-up molecular clustering measurements. The application of labelling antibodies therefore automatically introduce a localisation offset from the imaged protein of approximately ∼10 nm [103], a phenomenon known as the antibody ‘linkage error’.

This error can be somewhat minimised in post-processing analysis [73], by using smaller labelling nanobodies, or obviated by using different imaging systems. For instance, DNA-PAINT [55] (Figure 3) employs designed pairs of oligonucleotides, where fluorescently conjugated imaging stands controllably and transiently bind selected protein target previously labelled with complementary *docking* strands, with sub-10 nm localisation precision. In this instance, the transient binding of imaging stands represents blinking events that can be recorded over several thousand imaging frames, protected from photobleaching and representative of smaller structures when compared to both direct and indirect immunofluorescence.

In recent years, several adaptations to the DNA-PAINT system have been introduced. While some allow for brighter and faster DNA-PAINT imaging through the design of novel transient probes [104], others have improved the nanometric localisation accuracy of DNA-PAINT into the Ångström domain [105]. Employing either variation of DNA-PAINT for the study of GLUT4 at the PM would allow for a circumvention of the antibody linkage error seen in other SMLM imaging systems, with the added benefits of improved localisation accuracy and acquisition speed. Work is underway in our group to address this.

### Clusters or oligomers? Same or different?

Protein structural databases posit that many, if not most, proteins can form homo-oligomers [106]. Verification of this hypothesis is limited due of the challenging nature of the experimental approaches required. For instance, energy transfer methods such as fluorescence resonance energy transfer (FRET) or bioluminescence resonance energy transfer (BRET) have been employed to argue the close proximity of proteins [107,108]. However, these methods are limited as they cannot differentiate between true oligomers and molecules that are only ‘very close’ to each other [109], a limitation that is similarly applicable to all previously described super-resolution microscopy techniques (see above). Other microscopy based methods, to determine the oligomeric state of proteins, focus on the analysis of the temporal fluctuation of fluorescence intensity of a fluorescent protein of interest [110,111]. These approaches are challenging in the confines of PM-localised proteins.

G-protein coupled receptors (GPCR) are an example of a family of proteins that exist in different oligomeric states, which affects their function and in some cases even their location within the cell [109,112]. The dynamics of GPCR interactions have been studied using various approaches, including spatial intensity distribution analysis (SpIDA). In SpIDA, the protein of interest is tagged with a fluorescent protein and imaged using conventional confocal microscopy, in either live cells or fixed samples. From these images, the fluorescence intensity of the fluorescent protein is measured. On a conventional laser-scanning confocal microscope, this is usually achieved using an analogue photomultiplier tube that counts the number of collected photoelectrons [113]. The intensity of all pixels in a region of interest on the acquired image is then plotted as an intensity histogram over which a Poissonian distribution is fitted. This distribution will vary from monomeric, oligomeric, to a mixed populations and is used to calculate the quantal brightness ε of the fluorophore [114]. ε is defined as the mean intensity within a point spread function of a fluorescent unit [115]. The quantal brightness ε of a primarily monomeric population is defined as ε_0_. By reason, for a dimeric population ε will equal 2·ε_0_, 3·ε_0_ for a trimeric population, and so on. This approach has provided a powerful insight into the quaternary structure of different GPCR [116–119], and has for example revealed the antagonist-dependent oligomerisation behaviour of M_1_ muscarinic acetylcholine receptors [118].

We attempted to use this approach to examine the behaviour of GLUT4 tagged with EGFP (Silke Morris, unpublished work). While a trend towards a reduction in the oligomeric state of GLUT4 was observed, this did not reach significance. In addition, this analysis cannot distinguish between GLUT4 within the PM from GLUT4 within vesicles adjacent to the PM, hampering the utility of this approach.

### Fusion-with-retention and fusion-with-release – some speculations

A final question concerns what mechanism(s) may underscore the different GLUT4-vesicle exocytosis events alluded to above? The notion of a specialised subset of GLUT4-vesicles that fuses with the PM specifically in response to insulin has received a good deal of attention and experimental support as discussed above. However, as we and others have noted, the ability of insulin to promote increased levels of GLUT4 at the cell surface is found in a broad range of cells (including HeLa cells [120], podocytes [121] and neurones [122]), hinting toward more of a ubiquity in GLUT4 translocation machinery than initially thought [123].

**Figure 4:**
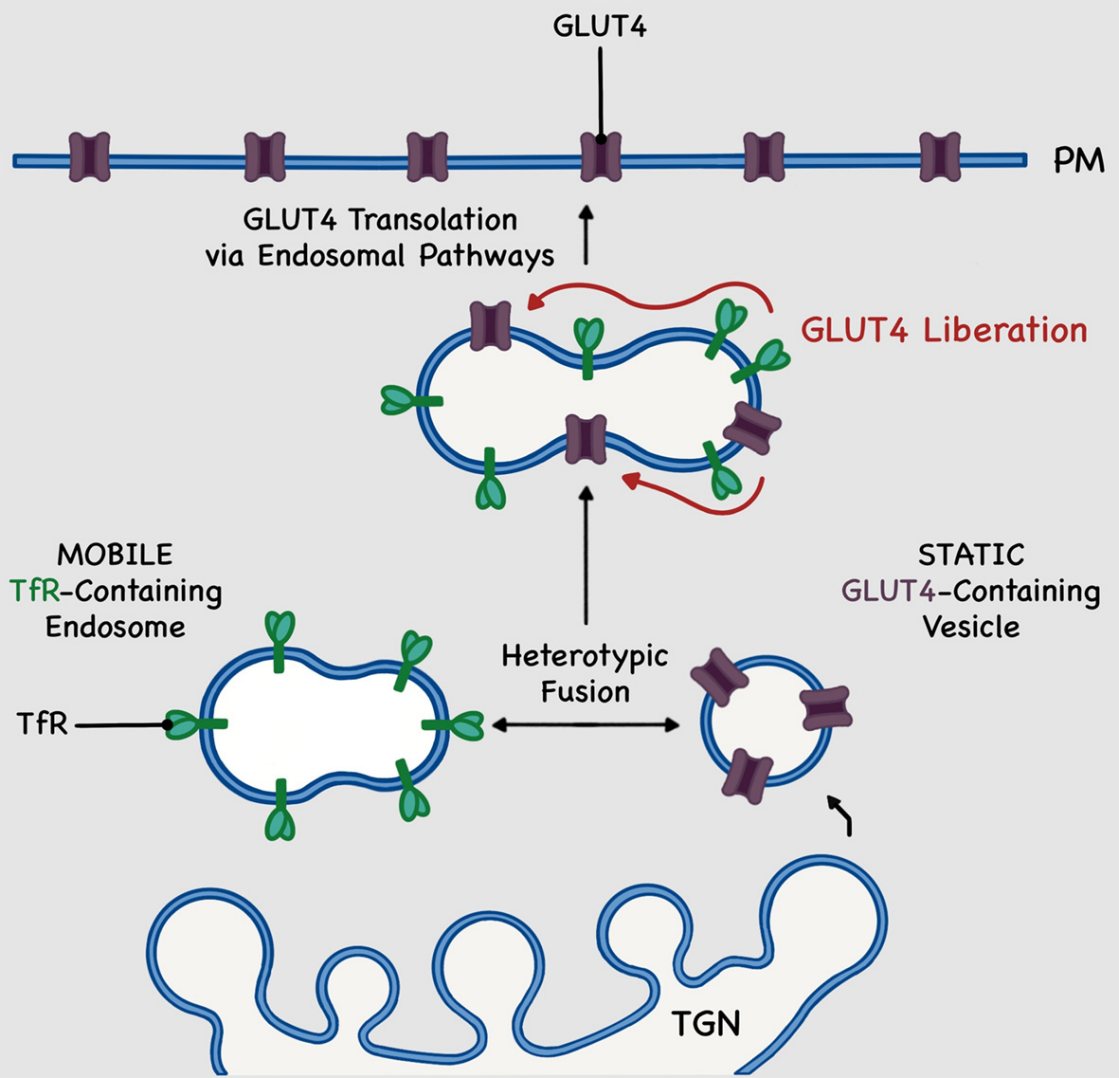
Endosomal-dependant model of insulin-stimulated GLUT4 trafficking to the plasma membrane Alternative model of GLUT4 trafficking to the plasma membrane via endosomal pathways. Insulin stimulates the heterotypic fusion of static GLUT4 (purple)-containing vesicles with mobile transferrin receptor (TfR; green)-containing endosomes in the *trans*-Golgi network (TGN) and perinuclear region. Upon entry into TfR-containing endosomes GLUT4 transporters are liberated from their static status and get dispersed amongst TfRs. In this newly formed compartment, GLUT4 translocate to the plasma membrane by taking advantage of the trafficking properties possessed by TfR-positive endosomes. This model differs from that shown in Figure 1 in the nature of the GLUT4 storage compartments and their subsequent mobilisation to the plasma membrane. This figure emphasises on a static intracellular pool of GLUT4 which, in the presence of insulin, reaches the cell surface via an endosomal pathway rather than a direct GLUT4-vesicle delivery. For details, refer to text and [117].

A recent study may offer some clues. By combining multiple types of quantitative analyses based on dual-colour live-cell imaging and super-resolution microscopy approaches (STED and TIRF-SIM) (Figure 3), Hatakeyama et al. recently provided new insights into GLUT4 trafficking events and suggested an alternative to the differential GLUT4-vesicle delivery model discussed above [67,124,125]. These studies focus on both GLUT4 and the TfR, a well-known recycling protein that, while insulin-responsive, exhibits a reduced extent of translocation compared with GLUT4. In 3T3-L1 adipocytes, Hatakeyama et al. documented anchoring mechanisms statically retaining GLUT4 in their storage compartments, close to TGN. Insulin was then shown to mediate the ‘heterotypic endosomal fusion’ of these static GLUT4-containing vesicles with mobile TfR-containing endosomes, followed by GLUT4 ‘liberation’, or individual movement, within these new compartments (Figure 4). As a result, the authors propose endosomal GLUT4 trafficking, rather than differential GLUT4-vesicle delivery as described previously, to the PM as a new insulin-responsive GLUT4 translocation paradigm [67,124,125]. This reflects to our earlier decision of using the term ‘GLUT4-vesicles’ rather than specific sub-population of GSC, highlighting the uncertainties surrounding the balance of these two mechanisms operating at any given point *in vivo*.

These models need not to be incompatible. It is entirely possible that in specialised cells, such as adipocytes and muscle cells, a portion of IRV which localise near the PM, are rapidly mobilised in response to insulin, as previously suggested [126]. After this initial ‘bolus’ of GLUT4 delivery, it is conceivable that other delivery mechanisms, such as those outlined in Figure 4 predominate. This is likely to be important as endocytosis will seek to re-internalise GLUT4 from the PM, unless it is rapidly re-exocytosed. It is also important to note that the experimental systems' extremes (no insulin or maximal insulin concentrations) do not represent physiological situations, and thus may bias interpretation and analysis of different mechanisms. The balance between these two delivery ‘pathways’ may perhaps underlie the proposed distinction in fusion-with-retention and fusion-with-release observations. Further work over a range of kinetic periods is clearly warranted.

## Conclusion

Understanding the dispersal mechanisms which control proteins within the plasma membrane represents a new frontier for cell biology and exemplifies the importance of interdisciplinary research. While this review highlights evidence in favour of GLUT4 dispersal in response to insulin, understanding the biological relevance of this mechanism and its operation in a range of cells, other proteins and disease, offers the potential for fascinating new cellular insight and potential new therapies.

## Open Access

Open access for this article was enabled by the participation of University of Strathclyde in an all-inclusive *Read & Publish* agreement with Portland Press and the Biochemical Society under a transformative agreement with JISC.

## Abbreviations

BRET: bioluminescence resonance energy transfer
DNA-PAINT: DNA-point accumulation in nanoscale topography
dSTORM: direct stochastic orientation reconstruction microscopy
FRET: fluorescence resonance energy transfer
GFP: green fluorescent protein
GPCR: G-protein coupled receptor
GSC: GLU4 storage compartments
HA: hemagglutinin
IRV: insulin responsive vesicles
LSM: light sheet microscopy
NA: numerical aperture
PALM: photo-activated localisation microscopy
PI4K-IIIα: phosphatidylinositol 4-kinase type IIIα
PI4P: phosphatidylinositol 4-phosphate
PM: plasma membrane
SIM: structured illumination microscopy
SOFI: super-resolution optical fluctuation imaging
SMLM: single molecule localisation microscopy
SpIDA: spatial intensity distribution analysis
SRRF: super-resolution radial fluctuations
STED: stimulated emission depletion
TfR: transferrin receptor
TGN: *trans*-Golgi network
TIRF: total internal reflection fluorescence

## References

[1] Mueckler M. and Thorens B. (2013) The SLC2 (GLUT) family of membrane transporters. Mol. Aspects Med. 34, 121–138 10.1016/j.mam.2012.07.001 23506862 PMC4104978

[2] Mueckler M., Caruso C., Baldwin S.A., Panico M., Blench I., Morris H.R. et al. (1985) Sequence and structure of a human glucose transporter. Science 229, 941–945 10.1126/science.3839598 3839598

[3] Bell G.I., Kayano T., Buse J.B., Burant C.F., Takeda J., Lin D. et al. (1990) Molecular biology of mammalian glucose transporters. Diabetes Care. 13, 198–208 10.2337/diacare.13.3.198 2407475

[4] Kayano T., Burant C.F., Fukumoto H., Gould G.W., Fan Y.S., Eddy R.L. et al. (1990) Human facilitative glucose transporters. Isolation, functional characterization, and gene localization of cDNAs encoding an isoform (GLUT5) expressed in small intestine, kidney, muscle, and adipose tissue and an unusual glucose transporter pseudogene-like sequence (GLUT6). J. Biol. Chem. 265, 13276–13282 10.1016/S0021-9258(19)38295-X 1695905

[5] Joost H.-G. and Thorens B. (2001) The extended GLUT-family of sugar/polyol transport facilitators: nomenclature, sequence characteristics, and potential function of its novel members. Mol. Membr. Biol. 18, 247–256 10.1080/09687680110090456 11780753

[6] Wu X. and Freeze H.H. (2002) GLUT14, a duplicon of GLUT3, is specifically expressed in testis as alternative splice forms. Genomics 80, 553–557 10.1006/geno.2002.7010 12504846

[7] Lundsgaard E. (1939) On the mode of action of insulin. Upsala Läk.-Fören. Förh. 45, 143

[8] Kruhøffer P. and Crone C. (1972) Einar Lundsgaard, 1899–1968. Ergeb. Physiol. 65, 1–14, 978-3-540-37462-6 10.1007/3-540-05814-1_1 4566422

[9] Wardzala L.J., Cushman S.W. and Salans L.B. (1978) Mechanism of insulin action on glucose transport in the isolated rat adipose cell. Enhancement of the number of functional transport systems. J. Biol. Chem. 253, 8002–8005 10.1016/S0021-9258(17)34350-8 711732

[10] Cushman S.W. and Wardzala L.J. (1980) Potential mechanism of insulin action on glucose transport in the isolated rat adipose cell. Apparent translocation of intracellular transport systems to the plasma membrane. J. Biol. Chem. 255, 4758–4762 10.1016/S0021-9258(19)85561-8 6989818

[11] Suzuki K. and Kono T. (1980) Evidence that insulin causes translocation of glucose transport activity to the plasma membrane from an intracellular storage site. Proc. Natl. Acad. Sci. U.S.A. 77, 2542–2545 10.1073/pnas.77.5.2542 6771756 PMC349437

[12] Wardzala L.J. and Jeanrenaud B. (1981) Potential mechanism of insulin action on glucose transport in the isolated rat diaphragm. Apparent translocation of intracellular transport units to the plasma membrane. J. Biol. Chem. 256, 7090–7093 10.1016/S0021-9258(19)68926-X 6265437

[13] Klip A., Ramlal T., Young D.A. and Holloszy J.O. (1987) Insulin‐induced translocation of glucose transporters in rat hindlimb muscles. FEBS Lett. 224, 224–230 10.1016/0014-5793(87)80452-0 2960560

[14] Bryant N.J., Govers R. and James D.E. (2002) Regulated transport of the glucose transporter GLUT4. Nat. Rev. Mol. Cell Biol. 3, 267–277 10.1038/nrm782 11994746

[15] James D.E., Brown R., Navarro J. and Pilch P.F. (1988) Insulin-regulatable tissues express a unique insulin-sensitive glucose transport protein. Nature 333, 183–185 10.1038/333183a0 3285221

[16] Charron M.J., Brosius F.C., Alper S.L. and Lodish H.F. (1989) A glucose transport protein expressed predominately in insulin-responsive tissues. Proc. Natl. Acad. Sci. U.S.A. 86, 2535–2539 10.1073/pnas.86.8.2535 2649883 PMC286951

[17] Fukumoto H., Kayano T., Buse J.B., Edwards Y., Pilch P.F., Bell G.I. et al. (1989) Cloning and characterization of the major insulin-responsive glucose transporter expressed in human skeletal muscle and other insulin-responsive tissues. J. Biol. Chem. 264, 7776–7779 10.1016/S0021-9258(18)83106-4 2656669

[18] Garcia de Herreros A. and Birnbaum M.J. (1989) The acquisition of increased insulin-responsive hexose transport in 3T3-L1 adipocytes correlates with expression of a novel transporter gene. J. Biol. Chem. 264, 19994–19999 10.1016/S0021-9258(19)47209-8 2479643

[19] Kaestner K.H., Christy R.J., McLenithan J.C., Braiterman L.T., Cornelius P., Pekala P.H. et al. (1989) Sequence, tissue distribution, and differential expression of mRNA for a putative insulin-responsive glucose transporter in mouse 3T3-L1 adipocytes. Proc. Natl. Acad. Sci. U.S.A. 86, 3150–3154 10.1073/pnas.86.9.3150 2654938 PMC287083

[20] Slot J.W., Geuze H.J., Gigengack S., Lienhard G.E. and James D.E. (1991) Immuno-localization of the insulin regulatable glucose transporter in brown adipose tissue of the rat. J. Cell Biol. 113, 123–135 10.1083/jcb.113.1.123 2007617 PMC2288909

[21] Klip A., McGraw T.E. and James D.E. (2019) Thirty sweet years of GLUT4. J. Biol. Chem. 294, 11369–11381 10.1074/jbc.REV119.008351 31175156 PMC6663870

[22] Gould G.W., Brodsky F.M. and Bryant N.J. (2020) Building GLUT4 Vesicles: CHC22 Clathrin’s Human Touch. Trends Cell Biol. 30, 705–719 10.1016/j.tcb.2020.05.007 32620516

[23] Slot J.W., Geuze H.J., Gigengack S., James D.E. and Lienhard G.E. (1991) Translocation of the glucose transporter GLUT4 in cardiac myocytes of the rat. Proc. Natl Acad. Sci. U.S.A. 88, 7815–7819 10.1073/pnas.88.17.7815 1881917 PMC52394

[24] Malide D., Ramm G., Cushman S.W. and Slot J.W. (2000) Immunoelectron microscopic evidence that GLUT4 translocation explains the stimulation of glucose transport in isolated rat white adipose cells. J. Cell Sci. 113, 4203–4210 10.1242/jcs.113.23.4203 11069765

[25] Bogan J.S. (2012) Regulation of Glucose Transporter Translocation in Health and Diabetes. Annu. Rev. Biochem. 81, 507–532 10.1146/annurev-biochem-060109-094246 22482906

[26] Saltiel A.R. (2021) Insulin signaling in health and disease. J. Clin. Investig. 131, e142241 10.1172/JCI142241 33393497 PMC7773347

[27] Bernhardt U., Carlotti F., Hoeben R.C., Joost H.-G. and Al-Hasani H. (2009) A dual role of the N-terminal FQQI motif in GLUT4 trafficking. Biol. Chem. 390, 883–892 10.1515/BC.2009.095 19558319

[28] Garvey W.T., Maianu L., Zhu J.H., Brechtel-Hook G., Wallace P. and Baron A.D. (1998) Evidence for defects in the trafficking and translocation of GLUT4 glucose transporters in skeletal muscle as a cause of human insulin resistance. J. Clin. Investig. 101, 2377–2386 10.1172/JCI1557 9616209 PMC508827

[29] Bogan J.S. and Kandror K.V. (2010) Biogenesis and regulation of insulin-responsive vesicles containing GLUT4. Curr. Opin. Cell Biol. 22, 506–512 10.1016/j.ceb.2010.03.012 20417083 PMC2910140

[30] Kahn B.B. (2019) Adipose Tissue, Inter-Organ Communication, and the Path to Type 2 Diabetes: The 2016 Banting Medal for Scientific Achievement Lecture. Diabetes 68, 3–14 10.2337/dbi18-0035 30573674 PMC6302542

[31] Santoro A., McGraw T.E. and Kahn B.B. (2021) Insulin action in adipocytes, adipose remodeling, and systemic effects. Cell Metab. 33, 748–757 10.1016/j.cmet.2021.03.019 33826917 PMC8078167

[32] Livingstone R., Bryant N.J., Boyle J.G., Petrie J.R. and Gould G.W. (2022) Diabetes is accompanied by changes in the levels of proteins involved in endosomal GLUT4 trafficking in obese human skeletal muscle. Endocrinol Diabetes Metab. 5, e361 10.1002/edm2.361 35964329 PMC9471587

[33] Shimomura O., Johnson F.H. and Saiga Y. (1962) Extraction, purification and properties of aequorin, a bioluminescent protein from the luminous hydromedusan, Aequorea. J. Cell. Comp. Physiol. 59, 223–239 10.1002/jcp.1030590302 13911999

[34] Tsien R.Y. (1998) The green fluorescent protein. Annu. Rev. Biochem. 509–544 10.1146/annurev.biochem.67.1.509 9759496

[35] Dobson S.P., Livingstone C., Gould G.W. and Tavaré J.M. (1996) Dynamics of insulin-stimulated translocation of GLUT4 in single living cells visualised using green fluorescent protein. FEBS Lett. 393, 179–184 10.1016/0014-5793(96)00879-4 8814285

[36] Dawson K., Aviles-Hernandez A., Cushman S.W. and Malide D. (2001) Insulin-regulated trafficking of dual-labeled glucose transporter 4 in primary rat adipose cells. Biochem. Biophys. Rep. 287, 445–454 10.1006/bbrc.2001.5620 11554749

[37] Fagerberg L., Jonasson K., von Heijne G., Uhlén M. and Berglund L. (2010) Prediction of the human membrane proteome. Proteomics 10, 1141–1149 10.1002/pmic.200900258 20175080

[38] Abbe E. (1873) Beiträge zur Theorie des Mikroskops und der mikroskopischen Wahrnehmung. Archiv für Mikroskopische Anatomie 1, 413–468 10.1007/BF02956173

[39] Huang B., Bates M. and Zhuang X. (2009) Super-resolution fluorescence microscopy. Annu. Rev. Biochem. 78, 993–1016 10.1146/annurev.biochem.77.061906.092014 19489737 PMC2835776

[40] Galbraith C.G. and Galbraith J.A. (2011) Super-resolution microscopy at a glance. J. Cell Sci. 124, 1607–1611 10.1242/jcs.080085 21536831 PMC3085433

[41] Schermelleh L., Ferrand A., Huser T., Eggeling C., Sauer M., Biehlmaier O. et al. (2019) Super-resolution microscopy demystified. Nat. Cell Biol. 21, 72–84 10.1038/s41556-018-0251-8 30602772

[42] Jacquemet G., Carisey A.F., Hamidi H., Henriques R. and Leterrier C. (2020) The cell biologist's guide to super-resolution microscopy. J. Cell Sci. 133, jcs240713 10.1242/jcs.240713 32527967

[43] Valli J., Garcia-Burgos A., Rooney L.M., Vale de Melo E.O.B., Duncan R.R. and Rickman C. (2021) Seeing beyond the limit: A guide to choosing the right super-resolution microscopy technique. J. Biol. Chem. 297, 100791 10.1016/j.jbc.2021.100791 34015334 PMC8246591

[44] Liu S., Hoess P. and Ries J. (2022) Super-resolution microscopy for structural cell biology. Annu. Rev. Biophys. 301–326 10.1146/annurev-biophys-102521-112912 35119945

[45] Axelrod D. (1981) Cell-substrate contacts illuminated by total internal reflection fluorescence. J. Cell Biol. 89, 141–145 10.1083/jcb.89.1.141 7014571 PMC2111781

[46] Gustafsson M.G.L. (2000) Surpassing the lateral resolution limit by a factor of two using structured illumination microscopy. J. Microsc. 198, 82–87 10.1046/j.1365-2818.2000.00710.x 10810003

[47] Chen B.-C., Legant W.R., Wang K., Shao L., Milkie D.E., Davidson M.W. et al. (2014) Lattice light-sheet microscopy: Imaging molecules to embryos at high spatiotemporal resolution. Science 346, 1257998 10.1126/science.1257998 25342811 PMC4336192

[48] Hell S.W. and Wichmann J. (1994) Breaking the diffraction resolution limit by stimulated emission: stimulated-emission-depletion fluorescence microscopy. Opt. Lett. 19, 780–782 10.1364/OL.19.000780 19844443

[49] Willig K.I., Harke B., Medda R. and Hell S.W. (2007) STED microscopy with continuous wave beams. Nat. Methods 4, 915–918 10.1038/nmeth1108 17952088

[50] Betzig E., Patterson G.H., Sougrat R., Lindwasser O.W., Olenych S., Bonifacino J.S. et al. (2006) Imaging intracellular fluorescent proteins at nanometer resolution. Science 313, 1642–1645 10.1126/science.1127344 16902090

[51] Rust M.J., Bates M. and Zhuang X. (2006) Sub-diffraction-limit imaging by stochastic optical reconstruction microscopy (STORM). Nat. Methods 3, 793–795 10.1038/nmeth929 16896339 PMC2700296

[52] Herdly L., Tinning P.W., Geiser A., Taylor H., Gould G.W. and van de Linde S. (2023) Benchmarking thiolate-driven photoswitching of cyanine dyes. J. Phys. Chem. B 127, 732–741 10.1021/acs.jpcb.2c06872 36638265 PMC9884076

[53] Heilemann M., van de Linde S., Schüttpelz M., Kasper R., Seefeldt B., Mukherjee A. et al. (2008) Subdiffraction-resolution fluorescence imaging with conventional fluorescent probes. Angew. Chem. Int. Ed. 47, 6172–6176 10.1002/anie.200802376 18646237

[54] van de Linde S., Löschberger A., Klein T., Heidbreder M., Wolter S., Heilemann M. et al. (2011) Direct stochastic optical reconstruction microscopy with standard fluorescent probes. Nat. Protoc. 6, 991–1009 10.1038/nprot.2011.336 21720313

[55] Schnitzbauer J., Strauss M.T., Schlichthaerle T., Schueder F. and Jungmann R. (2017) Super-resolution microscopy with DNA-PAINT. Nat. Protoc. 12, 1198–1228 10.1038/nprot.2017.024 28518172

[56] Jouchet P., Cabriel C., Bourg N., Bardou M., Poüs C., Fort E. et al. (2021) Nanometric axial localization of single fluorescent molecules with modulated excitation. Nat. Photonics 15, 297–304 10.1038/s41566-020-00749-9

[57] Gwosch K.C., Pape J.K., Balzarotti F., Hoess P., Ellenberg J., Ries J. et al. (2020) MINFLUX nanoscopy delivers 3D multicolor nanometer resolution in cells. Nat. Methods 17, 217–224 10.1038/s41592-019-0688-0 31932776

[58] Dertinger T., Colyer R., Iyer G., Weiss S. and Enderlein J. (2009) Fast, background-free, 3D super-resolution optical fluctuation imaging (SOFI). Proc. Natl Acad. Sci. U.S.A. 106, 22287–22292 10.1073/pnas.0907866106 20018714 PMC2799731

[59] Gustafsson N., Culley S., Ashdown G., Owen D.M., Pereira P.M. and Henriques R. (2016) Fast live-cell conventional fluorophore nanoscopy with ImageJ through super-resolution radial fluctuations. Nat. Commun. 7, 12471 10.1038/ncomms12471 27514992 PMC4990649

[60] Li C.H., Bai L., Li D.D., Xia S. and Xu T. (2004) Dynamic tracking and mobility analysis of single GLUT4 storage vesicle in live 3T3-L1 cells. Cell Res. 14, 480–486 10.1038/sj.cr.7290251 15625015

[61] Lizunov V.A., Matsumoto H., Zimmerberg J., Cushman S.W. and Frolov V.A. (2005) Insulin stimulates the halting, tethering, and fusion of mobile GLUT4 vesicles in rat adipose cells. J. Cell Biol. 169, 481–489 10.1083/jcb.200412069 15866888 PMC2171949

[62] Jiang L., Fan J., Bai L., Wang Y., Chen Y., Yang L. et al. (2008) Direct quantification of fusion rate reveals a distal role for AS160 in insulin-stimulated fusion of GLUT4 storage vesicles. J. Biol. Chem. 13, 8508–8516 10.1074/jbc.M708688200 PMC2417169 18063571

[63] Lizunov V.A., Lisinski I., Stenkula K., Zimmerberg J. and Cushman S.W. (2009) Insulin regulates fusion of GLUT4 vesicles independent of Exo70-mediated tethering. J. Biol. Chem. 284, 7914–7919 10.1074/jbc.M806460200 19155211 PMC2658084

[64] Bai L., Wang Y., Fan J., Chen Y., Ji W., Qu A. et al. (2007) Dissecting multiple steps of GLUT4 trafficking and identifying the sites of insulin action. Cell Metab. 5, 47–57 10.1016/j.cmet.2006.11.013 17189206

[65] Fujita H., Hatakeyama H., Watanabe T.M., Sato M., Higuchi H. and Kanzaki M. (2010) Identification of three distinct functional sites of insulin-mediated GLUT4 trafficking in adipocytes using quantitative single molecule imaging. Mol. Biol. Cell 21, 2721–2731 10.1091/mbc.e10-01-0029 20519436 PMC2912357

[66] Sun Y., Jaldin-Fincati J., Liu Z., Bilan P.J. and Klip A. (2016) A complex of Rab13 with MICAL-L2 and α-actinin-4 is essential for insulin-dependent GLUT4 exocytosis. Mol. Biol. Cell 27, 75–89 10.1091/mbc.E15-05-0319 26538022 PMC4694764

[67] Hatakeyama H. and Kanzaki M. (2017) Heterotypic endosomal fusion as an initial trigger for insulin-induced glucose transporter 4 (GLUT4) translocation in skeletal muscle. J. Physiol. 595, 5603–5621 10.1113/JP273985 28556933 PMC5556175

[68] Richter V., Lanzerstorfer P., Weghuber J. and Schneckenburger H. (2020) Super-resolution live cell microscopy of membrane-proximal fluorophores. Int. J. Mol. Sci. 21, 7099 10.3390/ijms21197099 32993061 PMC7582769

[69] Stenkula K.G., Lizunov V.A., Cushman S.W. and Zimmerberg J. (2010) Insulin controls the spatial distribution of GLUT4 on the cell surface through regulation of its postfusion dispersal. Cell Metab. 12, 250–259 10.1016/j.cmet.2010.08.005 20816091 PMC3427691

[70] Holman G.D., Lo Leggio L. and Cushman S.W. (1994) Insulin-stimulated GLUT4 glucose transporter recycling. A problem in membrane protein subcellular trafficking through multiple pools. J. Biol. Chem. 269, 17516–17524 10.1016/S0021-9258(17)32471-7 8021259

[71] Lizunov V.A., Stenkula K., Troy A., Cushman S.W. and Zimmerberg J. (2013) Insulin regulates Glut4 confinement in plasma membrane clusters in adipose cells. PloS ONE 8, e57559 10.1371/journal.pone.0057559 23520472 PMC3592853

[72] Gao L., Chen J., Gao J., Wang H. and Xiong W. (2017) Super-resolution microscopy reveals the insulin-resistance-regulated reorganization of GLUT4 on plasma membranes. J. Cell Sci. 130, 396–405 27888215 10.1242/jcs.192450

[73] Koester A.M., Geiser A., Laidlaw K.M.E., Morris S., Cutiongco M.F.A., Stirrat L. et al. (2022) EFR3 and phosphatidylinositol 4-kinase IIIα regulate insulin-stimulated glucose transport and GLUT4 dispersal in 3T3-L1 adipocytes. Biosci. Rep. 42, 7 BSR20221181 10.1042/BSR20221181 35735144 PMC9272592

[74] Koester A.M., Geiser A., Bowman P.R.T., van de Linde S., Gadegaard N., Bryant N.J. et al. (2022) GLUT4 translocation and dispersal operate in multiple cell types and are negatively correlated with cell size in adipocytes. Sci. Rep. 12, 20535 10.1038/s41598-022-24736-y 36446811 PMC9708847

[75] Wollman A.J.M., Kioumourtzoglou D., Ward R., Gould G.W. and Bryant N.J. (2022) Large scale, single-cell FRET-based glucose uptake measurements within heterogeneous populations. iScience 25, 104023 10.1016/j.isci.2022.104023 35313696 PMC8933717

[76] Stenkula K.G. and Erlanson-Albertsson C. (2018) Adipose cell size: importance in health and disease. Am. J. Physiol. - Regul. 315, R284–R295 10.1152/ajpregu.00257.2017 29641234

[77] Shewan A.M., McCann R.K., Lamb C.A., Stirrat L., Kioumourtzoglou D., Adamson I.S. et al. (2013) Endosomal sorting of GLUT4 and Gap1 is conserved between yeast and insulin-sensitive cells. J. Cell Sci. 126, 1576–1582 10.1242/jcs.114371 23424197 PMC3647436

[78] Wieczorke R., Dlugai S., Krampe S. and Boles E. (2003) Characterisation of mammalian GLUT glucose transporters in a heterologous yeast expression system. Cell. Physiol. Biochem. 13, 123–134 10.1159/000071863 12876383

[79] Kasahara T. and Kasahara M. (1997) Characterization of rat Glut4 glucose transporter expressed in the yeast Saccharomyces cerevisiae: comparison with Glut1 glucose transporter. Biochim. Biophys. Acta Biomembr. 1324, 111–119 10.1016/S0005-2736(96)00217-9 9059504

[80] Schmidl S., Tamayo Rojas S.A., Iancu C.V., Choe J.Y. and Oreb M. (2020) Functional Expression of the Human Glucose Transporters GLUT2 and GLUT3 in Yeast Offers Novel Screening Systems for GLUT-Targeting Drugs. Front. Mol. Biosci. 7, 598419 10.3389/fmolb.2020.598419 33681287 PMC7930720

[81] Baird D., Stefan C., Audhya A., Weys S. and Emr S.D. (2008) Assembly of the PtdIns 4-kinase Stt4 complex at the plasma membrane requires Ypp1 and Efr3. J. Cell Biol. 183, 1061–1074 10.1083/jcb.200804003 19075114 PMC2600738

[82] Nakatsu F., Baskin J.M., Chung J., Tanner L.B., Shui G., Lee S.Y. et al. (2012) PtdIns4P synthesis by PI4KIIIalpha at the plasma membrane and its impact on plasma membrane identity. J. Cell Biol. 199, 1003–1016 10.1083/jcb.201206095 23229899 PMC3518224

[83] Bojjireddy N., Guzman-Hernandez M.L., Reinhard N.R., Jovic M. and Balla T. (2015) EFR3s are palmitoylated plasma membrane proteins that control responsiveness to G-protein-coupled receptors. J. Cell Sci. 128, 118–128 25380825 10.1242/jcs.157495 PMC4282049

[84] Waring M.J., Andrews D.M., Faulder P.F., Flemington V., McKelvie J.C., Maman S. et al. (2014) Potent, selective small molecule inhibitors of type III phosphatidylinositol-4-kinase α- but not β-inhibit the phosphatidylinositol signaling cascade and cancer cell proliferation. Chem. Comm. 50, 5388–5390 10.1039/C3CC48391F 24366037

[85] Adhikari H., Kattan W.E., Kumar S., Zhou P., Hancock J.F. and Counter C.M. (2021) Oncogenic KRAS is dependent upon an EFR3A-PI4KA signaling axis for potent tumorigenic activity. Nat. Commun. 12, 5248 10.1038/s41467-021-25523-5 34504076 PMC8429657

[86] Fujiwara T.K., Tsunoyama T.A., Takeuchi S., Kalay Z., Nagai Y., Kalkbrenner T. et al. (2023) Ultrafast single-molecule imaging reveals focal adhesion nano-architecture and molecular dynamics. J. Cell Biol. 222, e202110162 10.1083/jcb.202110162 37278764 PMC10244807

[87] Kasai R.S. and Kusumi A. (2014) Single-molecule imaging revealed dynamic GPCR dimerization. Curr. Opin. Cell Biol. 27, 78–86 10.1016/j.ceb.2013.11.008 24480089

[88] Sych T., Levental K.R. and Sezgin E. (2022) Lipid-protein interactions in plasma membrane organization and function. Annu. Rev. Biophys. 51, 135–156 10.1146/annurev-biophys-090721-072718 34982570 PMC12101515

[89] Levental I. and Lyman E. (2023) Regulation of membrane protein structure and function by their lipid nano-environment. Nat. Rev. Mol. Cell Biol. 24, 107–122 10.1038/s41580-022-00524-4 36056103 PMC9892264

[90] Jaqaman K., Kuwata H., Touret N., Collins R., Trimble W.S., Danuser G. et al. (2011) Cytoskeletal control of CD36 diffusion promotes its receptor and signaling function. Cell 146, 593–606 10.1016/j.cell.2011.06.049 21854984 PMC3160624

[91] Trimble W.S. and Grinstein S. (2015) Barriers to the free diffusion of proteins and lipids in the plasma membrane. J. Cell Biol. 208, 259–271 10.1083/jcb.201410071 25646084 PMC4315255

[92] Kusumi A., Fujiwara T.K., Tsunoyama T.A., Kasai R.S., Liu A.A., Hirosawa K.M. et al. (2020) Defining raft domains in the plasma membrane. Traffic 21, 106–137 10.1111/tra.12718 31760668

[93] Godó S., Barabás K., Lengyel F., Ernszt D., Kovács T., Kecskés M. et al. (2021) Single-molecule imaging reveals rapid estradiol action on the surface movement of AMPA receptors in live neurons. Front. Cell Dev. Biol. 9, 708715 10.3389/fcell.2021.708715 34631701 PMC8495425

[94] Yan Q., Lu Y., Zhou L., Chen J., Xu H., Cai M. et al. (2018) Mechanistic insights into GLUT1 activation and clustering revealed by super-resolution imaging. Proc. Natl. Acad. Sci. U.S.A. 115, 7033–7038 10.1073/pnas.1803859115 29915035 PMC6142262

[95] Diamond D.L. and Carruthers A. (1993) Metabolic control of sugar transport by derepression of cell surface glucose transporters. An insulin-independent recruitment-independent mechanism of regulation. J. Biol. Chem. 268, 6437–6444 10.1016/S0021-9258(18)53271-3 8454616

[96] Coderre P.E., Cloherty E.K., Zottola R.J. and Carruthers A. (1995) Rapid substrate translocation by the multisubunit, erythroid glucose transporter requires subunit associations but not cooperative ligand binding. Biochem 34, 9762–9773 10.1021/bi00030a014 7626647

[97] Cloherty E.K., Levine K.B. and Carruthers A. (2001) The red blood cell glucose transporter presents multiple, nucleotide-sensitive sugar exit sites. Biochem 40, 15549–15561 10.1021/bi015586w 11747430

[98] Raja M. and Kinne R.K.H. (2020) Mechanistic insights into protein stability and self-aggregation in GLUT1 genetic variants causing GLUT1-deficiency syndrome. J. Membr. Biol. 253, 87–99 10.1007/s00232-020-00108-3 32025761 PMC7150661

[99] van de Linde S. (2019) Single-molecule localization microscopy analysis with ImageJ. J. Phys. D: Appl. Phys. 52, 203002 10.1088/1361-6463/ab092f

[100] Dall'Agnese A., Platt J.M., Zheng M.M., Friesen M., Dall'Agnese G., Blaise A.M. et al. (2022) The dynamic clustering of insulin receptor underlies its signaling and is disrupted in insulin resistance. Nat. Commun. 13, 7522 10.1038/s41467-022-35176-7 36473871 PMC9727033

[101] Ditlev J.A., Vega A.R., Köster D.V., Su X., Tani T., Lakoduk A.M. et al. (2019) A composition-dependent molecular clutch between T cell signaling condensates and actin. eLife 8, e42695 10.7554/eLife.42695 31268421 PMC6624021

[102] Umebayashi M., Takemoto S., Reymond L., Sundukova M., Hovius R., Bucci A. et al. (2022) A covalently linked probe to monitor local membrane properties surrounding plasma membrane proteins. J. Cell Biol. 222, e202206119 10.1083/jcb.202206119 36571579 PMC9802683

[103] Ries J., Kaplan C., Platonova E., Eghlidi H. and Ewers H. (2012) A simple, versatile method for GFP-based super-resolution microscopy via nanobodies. Nat. Methods 9, 582–584 10.1038/nmeth.1991 22543348

[104] Chung K.K.H., Zhang Z., Kidd P., Zhang Y., Williams N.D., Rollins B. et al. (2022) Fluorogenic DNA-PAINT for faster, low-background super-resolution imaging. Nat. Methods 19, 554–559 10.1038/s41592-022-01464-9 35501386 PMC9133131

[105] Reinhardt S.C.M., Masullo L.A., Baudrexel I., Steen P.R., Kowalewski R., Eklund A.S. et al. (2023) Ångström-resolution fluorescence microscopy. Nature 617, 711–716 10.1038/s41586-023-05925-9 37225882 PMC10208979

[106] Henrick K. and Thornton J.M. (1998) PQS: a protein quaternary structure file server. Trends Biochem. 23, 358–361 10.1016/S0968-0004(98)01253-5 9787643

[107] Issafras H., Angers S., Bulenger S., Blanpain C., Parmentier M., Labbé-Jullié C. et al. (2002) Constitutive Agonist-independent CCR5 Oligomerization and Antibody-mediated Clustering Occurring at Physiological Levels of Receptors. J. Biol. Chem. 277, 34666–34673 10.1074/jbc.M202386200 12089144

[108] Floyd D.H., Geva A., Bruinsma S.P., Overton M.C., Blumer K.J. and Baranski T.J. (2003) C5a receptor oligomerization. II. Fluorescence resonance energy transfer studies of a human G protein-coupled receptor expressed in yeast. J. Biol. Chem. 278, 35354–35361 10.1074/jbc.M305607200 12835318

[109] Gurevich V.V. and Gurevich E.V. (2008) GPCR monomers and oligomers: it takes all kinds. Trends Neurosci. 31, 74–81 10.1016/j.tins.2007.11.007 18199492 PMC2366802

[110] Chen Y., Müller J.D., So P.T. and Gratton E. (1999) The photon counting histogram in fluorescence fluctuation spectroscopy. Biophys. J. 77, 553–567 10.1016/S0006-3495(99)76912-2 10388780 PMC1300352

[111] Kask P., Palo K., Ullmann D. and Gall K. (1999) Fluorescence-intensity distribution analysis and its application in biomolecular detection technology. Proc. Natl Acad. Sci. U.S.A. 96, 13756–13761 10.1073/pnas.96.24.13756 10570145 PMC24137

[112] Fotiadis D., Jastrzebska B., Philippsen A., Müller D.J., Palczewski K. and Engel A. (2006) Structure of the rhodopsin dimer: a working model for G-protein-coupled receptors. Curr. Opin. Struct. Biol. 16, 252–259 10.1016/j.sbi.2006.03.013 16567090

[113] Paddock S.W. (1999) Confocal laser scanning microscopy. BioTechniques 27, 992–1004 10.2144/99275ov01 10572648

[114] Godin A.G., Costantino S., Lorenzo L.-E., Swift J.L., Sergeev M., Ribeiro-da-Silva A. et al. (2011) Revealing protein oligomerization and densities in situ using spatial intensity distribution analysis. Proc. Natl. Acad. Sci. U.S.A. 108, 7010–7015 10.1073/pnas.1018658108 21482753 PMC3084122

[115] Godin A.G., Rappaz B., Potvin-Trottier L., Kennedy T.E., De Koninck Y. and Wiseman P.W. (2015) Spatial intensity distribution analysis reveals abnormal oligomerization of proteins in single cells. Biophysi. J. 109, 710–721 10.1016/j.bpj.2015.06.068 PMC4547338 26287623

[116] Zakrys L., Ward R.J., Pediani J.D., Godin A.G., Graham G.J. and Milligan G. (2014) Roundabout 1 exists predominantly as a basal dimeric complex and this is unaffected by binding of the ligand Slit2. Biochem. J. 461, 61–73 10.1042/BJ20140190 24673457

[117] Ward R.J., Pediani J.D., Godin A.G. and Milligan G. (2015) Regulation of oligomeric organization of the serotonin 5-hydroxytryptamine 2C (5-HT2C) receptor observed by spatial intensity distribution analysis. J. Biol. Chem. 290, 12844–12857 10.1074/jbc.M115.644724 25825490 PMC4432300

[118] Pediani J.D., Ward R.J., Godin A.G., Marsango S. and Milligan G. (2016) Dynamic regulation of quaternary organization of the M1 muscarinic receptor by subtype-selective antagonist drugs. J. Biol. Chem. 291, 13132–13146 10.1074/jbc.M115.712562 27080256 PMC4933229

[119] Marsango S., Caltabiano G., Jiménez-Rosés M., Millan M.J., Pediani J.D., Ward R.J. et al. (2017) A molecular basis for selective antagonist destabilization of dopamine D3 receptor quaternary organization. Sci. Rep. 7, 2134 10.1038/s41598-017-02249-3 28522847 PMC5437050

[120] Morris S., Geoghegan N.D., Sadler J.B.A., Koester A.M., Black H.L., Laub M. et al. (2020) Characterisation of GLUT4 trafficking in HeLa cells: comparable kinetics and orthologous trafficking mechanisms to 3T3-L1 adipocytes. PeerJ 8, e8751 10.7717/peerj.8751 32185116 PMC7060922

[121] Tolvanen T.A., Dash S.N., Polianskyte-Prause Z., Dumont V. and Lehtonen S. (2015) Lack of CD2AP disrupts Glut4 trafficking and attenuates glucose uptake in podocytes. J. Cell Sci. 128, 4588–4600 10.1242/jcs.175075 26546360

[122] Ashrafi G., Wu Z., Farrell R.J. and Ryan T.A. (2017) GLUT4 mobilization supports energetic demands of active synapses. Neuron 93, 606.e603–615.e603 10.1016/j.neuron.2016.12.020 28111082 PMC5330257

[123] Bryant N.J. and Gould G.W. (2020) Insulin stimulated GLUT4 translocation – Size is not everything!. Curr. Opin. Cell Biol. 65, 28–34 10.1016/j.ceb.2020.02.006 32182545

[124] Hatakeyama H. and Kanzaki M. (2011) Molecular basis of insulin-responsive GLUT4 trafficking systems revealed by single molecule imaging. Traffic 12, 1805–1820 10.1111/j.1600-0854.2011.01279.x 21910807

[125] Hatakeyama H., Kobayashi K. and Kanzaki M. (2022) Three live-imaging techniques for comprehensively understanding the initial trigger for insulin-responsive intracellular GLUT4 trafficking. iScience 25, 104164 10.1016/j.isci.2022.104164 35434546 PMC9010770

[126] Kioumourtzoglou D., Gould G.W. and Bryant N.J. (2014) Insulin stimulates syntaxin4 SNARE complex assembly via a novel regulatory mechanism. Mol. Cell. Biol. 34, 1271–1279 10.1128/MCB.01203-13 24469400 PMC3993566

